# Fibers-based temporal super-resolved imaging

**DOI:** 10.1038/s41598-020-74879-z

**Published:** 2020-10-20

**Authors:** Sagie Asraf, Moti Fridman, Zeev Zalevsky

**Affiliations:** Faculty of Engineering and the Institute for Nanotechnology and Advanced Materials Bar-Ilan University, 52900 Ramat-Gan, Israel

**Keywords:** Optical techniques, Fibre optics and optical communications

## Abstract

In this paper we present a new technique for a fiber-based temporal super resolving system allowing to improve the resolution of a temporal imaging system. The proposed super resolving concept is based upon translating the field of view multiplexing method that is used to increase resolution in spatial imaging systems from the spatial domain to the temporal domain. In this paper, an optical realization of our proposed system is presented, using optical fibers and electro-optic modulators. In addition, we show how one can apply this method using low-rate electronics for the required modulation. We also show simulation results that demonstrate the high resolution accepted in our method compares to the basic temporal imaging system. Experimental results which demonstrate resolution improvement by a factor of 1.5 based on the proposed method are presented together with an additional experiment that shows the ability to generate the desired modulation with low rate electronics.

## Introduction

The topic of spatial super-resolved imaging has been highly investigated both from a scientific point of view as well as due to its applicability based prospective. There are various super resolving configurations developed in the field of spatial optical imaging. Generally, the idea is always to encode the spatial data in a way that will convert the high spatial resolution to some other domains such as time, wavelength, polarization, etc., then one can transmit the encoded data via the resolution limited imaging system and eventually to perform digital decoding^1^. The process of encoding is also called multiplexing and many configurations were developed to present time-multiplexing^2–4^, wavelength multiplexing^5^, polarization multiplexing^6–8^, field of view (FOV) multiplexing^9–11^, and more. The field of transforming data processing capabilities from the spatial domain towards the time domain has also become common due to the simplicity in converting the temporal spectrum into space^12,13^.

The analogy between the diffraction of light in space and dispersion of pulses in time has been known for many years^14,15^. This analogy led to the development of a time lens, which is an optical component that enables to extend and compress temporal optical signals while preserving its overall profile^16–18^, and so it enables to extend events in time and investigating them more efficiently. An optical time-lens may be realized in different ways such as by using the four-wave mixing (FWM) effect^19,20^, ultra-short temporal shutter which acts as a temporal pinhole camera^21,22^, or by using an electro-optic phase modulator (EOM)^16,18^.

Since there is an analogy between spatial imaging systems and temporal imaging systems, the optical time-lens has resolution limitation associated due to its limited temporal size similar to the resolution limitation of the spatial lens. Spatial resolution limitation was first analyzed by Ernest Abbe^23^ to be proportional to the optical wavelength and the F-number of the imaging system and was later mathematically supported by Lord Rayleigh^24^. Based on a similar concept, the temporal resolution was defined in previous works to be the shortest transform-limited Gaussian FWHM pulse-width that we get in the output of the lens after inserting a Gaussian pulse in it^25^. Because of this resolution limitation, temporal super-resolution methods have been proposed such as a method that is based on localization microscopy algorithm^26^, time lens array imaging^27^, and a method that is inspired by structured illumination microscopy^28^.

Because of the similarity between time and space, similar super resolving concepts being so nicely implemented in the spatial domain can be adapted to the time domain. However, usage of time multiplexing super-resolution concepts for temporal signals^29^ might be limited for fundamental reasons and polarization multiplexing might be problematic for realization since fiber-optics systems are commonly using fibers which do not maintain the polarization state. Thus, one of the most suitable super resolving concepts as being well-matched to the time domain might be the FOV multiplexing super-resolution approach, which using fixed generalized gratings in predetermined positions^9^. Note that applying this concept in the case of spatial imaging is possible both when using coherent as well as an incoherent type of illumination.

In this manuscript, we will show how one can adapt the FOV multiplexing super resolving concept of Ref.^9^ from the space to the temporal imaging domain. For that purpose, we use temporal gratings which are the temporal analog to the diffraction optical grating in the spatial case. Similar to the space grating, that generates a replica of the input field in the spatial frequency domain, the temporal grating generates a spectral replica of the temporal signal in the frequency domain^30^. It was shown that such gratings can be realized by using electro-optic intensity modulators^30,31^. The full description of our proposed super-resolved imaging system will be presented in the Methods section, along with a detailed explanation of how to implement the temporary gratings using low-electronics devices and integrate them into the proposed system. Then we show simulation results that demonstrate the high resolution accepted in our method compares to the basic temporal imaging system. This paper also presents preliminary experimental results which demonstrate resolution improvement by a factor of 1.5 based on the proposed method, and additional experimental results that show the ability to generate desired temporal grating with low-rate electronics. Finally, we will discuss the meaning of the obtained results and future possible research directions.

## Theory

### The proposed super-resolution imaging system

**Figure 1:**
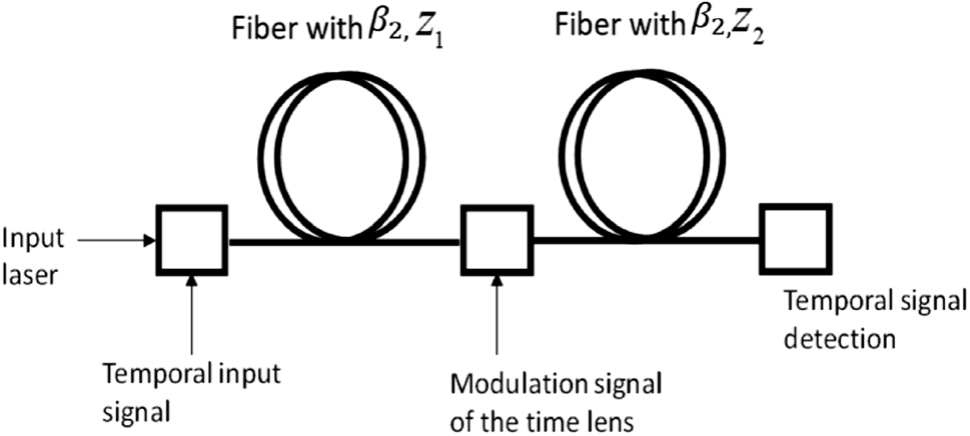
Schematic sketch of the regular optical imaging system.

We first introduce the basic configuration for the temporal imaging system using a temporal lens based on an electro-optic phase modulator. The system consists of dispersion fiber with a length of $z_{1}$, an electro-optic modulator (EOM) driven with a sinusoidal voltage with a radial frequency of $\omega_{m}$ and additional dispersion fiber with a length of $z_{2}$^16^. The schematic diagram of this system can be seen in Fig. 1.

If the optical waveform of the input is shorter than $1/\omega_{m}$, then the phase modulation of the modulator is essentially quadratic under any extremum of the sinusoid.

1 $$ \emptyset \left( t \right) = \pm A\left( {1 - \frac{{\omega_{m}^{2} t^{2} }}{2}} \right) $$

where A is the modulation index of the phase modulator. In this case, the temporal phase modulation of the signal is equivalent to the phase accumulation of spatial signal where it passes through a spatial lens. Since there is an analogy between propagation through the fiber and free-space propagation, the above-illustrated setup is the temporal equivalence of spatial imaging system. Thus, we can define the imaging condition for this system similar to the spatial case by^16^:

$$ \frac{1}{{z_{1} }} + \frac{1}{{z_{2} }} = A\omega_{m}^{2} \beta_{2} $$

where $\beta_{2}$ is the second-order dispersion coefficient of the optical fibers. Similarly, we can extract from Eq. 2 the magnification (i.e. scaling in the time domain) of this temporal imaging system by finding the ratio between the distances $z_{1}$ and $z_{2}$:

$$ M = \left| {A\omega_{m}^{2} \beta_{2} z_{2} - 1} \right| = {\raise0.7ex\hbox{${z2}$} \!\mathord{\left/ {\vphantom {{z2} {z1}}}\right.\kern-\nulldelimiterspace} \!\lower0.7ex\hbox{${z1}$}} $$

**Figure 2:**
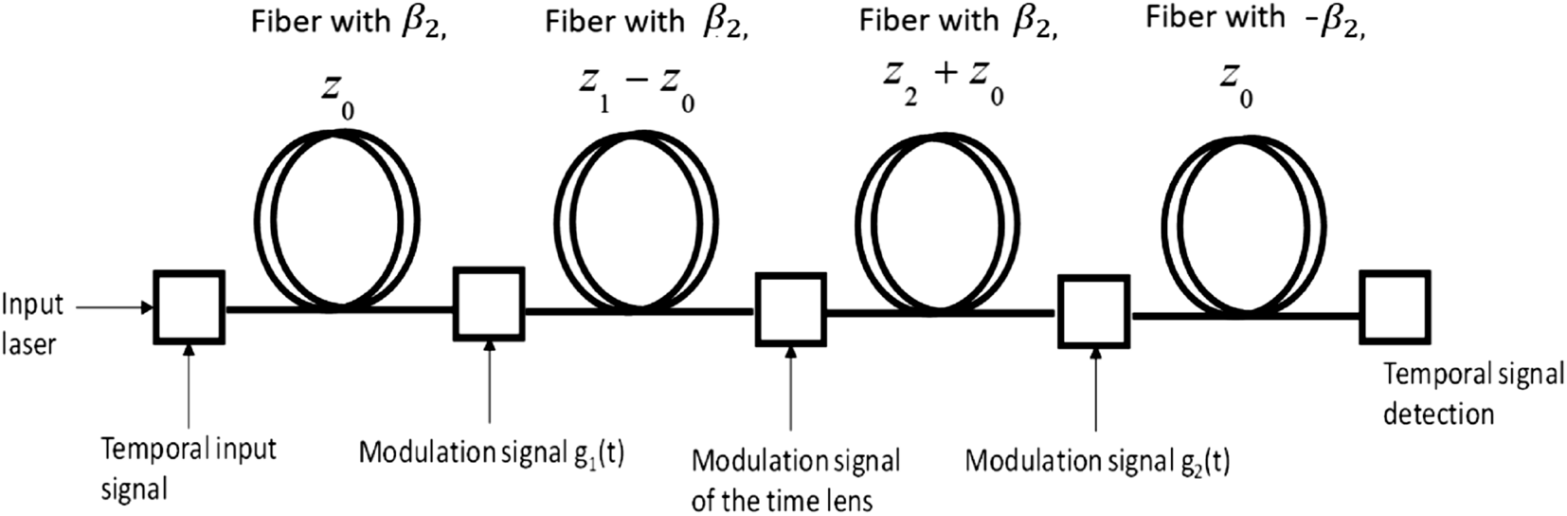
Schematic sketch of the proposed super resolving scheme.

The temporal length of this time lens (equivalent to the size of a spatial lens) equals at most to $1/\omega_{m}$^16^ since the quadratic approximation of the phase modulation is true only for such short pulses. This finite temporal aperture limiting the temporal resolution of this imaging system. To overcome this resolution limitation, the super-resolved imaging system follows the schematic sketch appearing in Fig. 2. is proposed.

As can be seen in Fig. 2 the proposed system consists of a dispersive fiber with a distance of $z_{0}$, temporal grating, the basic configuration of the temporal imaging system as illustrated in Fig. 1, another temporal grating and another dispersive fiber with a distance of $z_{0}$ but with negative dispersion coefficient − $\beta_{2}$. The temporal gratings denoted by $g_{1}$ and $g_{2}$, realized by using electro-optic intensity modulators, are periodic functions with a radial frequency of $\omega_{g}$. The mathematical formalism of those gratings can be decomposed according to Fourier series decomposition as:

$$ g_{1} \left( t \right) = \mathop \sum \limits_{n} C_{n} e^{{in\omega_{g} t}} $$

$$ g_{2} \left( t \right) = \mathop \sum \limits_{m} B_{m} e^{{im\omega_{g} t}} $$

By using the analogy between spatial imaging and temporal imaging, the propagation of the temporal input signal through all the above- illustrated setup can be mathematically formulated. The full mathematical development describes this signal's propagation is shown in the first part of the Supplementary document of this paper. The accepted output signal will be:

5 $$ u\left( {\omega_{0} ,t,z_{out} } \right) = u_{0} e^{{i\omega_{0} t - ik_{z,,0} z_{out} }} \mathop \sum \limits_{n} \mathop \sum \limits_{m} B_{m} C_{n} \mathop \smallint \nolimits \tilde{U}\left( {\omega - \omega_{0} } \right)rect\left( {\frac{{\omega - \omega_{0} + n\omega_{g} }}{{\omega_{m} }}} \right)e^{{i\frac{{\beta_{2} z_{0} }}{2}\left( {\omega - \omega_{0} } \right)^{2} }} \ldots e^{{ - i\frac{{\beta_{2} z_{0} }}{2}\left( {\omega - \omega_{0} + \omega_{g} \left( {n + m} \right)} \right)^{2} }} e^{{i\left( {t - \frac{{z_{out} }}{{v_{g} }}} \right)\left( {\omega - \omega_{0} + \omega_{g} \left( {n + m} \right)} \right)}} d\left( {\omega - \omega_{0} } \right) $$

where $\tilde{U}(\omega - \omega_{0} )$ is the Fourier transform of the overall temporal signal, which includes temporal low-frequency modulation signal carried on top of an optical carrier $\omega_{0}$, $v_{g}$ is the group velocity and $k_{z,0}$ is the wavenumber for $\omega = \omega_{0}$. To obtain super-resolution we wish to have (n + m) = 0. If this condition is fulfilled, then we obtain the output expression to be:

6 $$ u\left( {\omega_{0} ,t,z_{out} } \right) = u_{0} e^{{i\omega_{0} t - ik_{zo} z_{out} }} \mathop \sum \limits_{n} B_{ - n} C_{n} \mathop \smallint \nolimits \tilde{U}\left( {\omega - \omega_{0} } \right)rect\left( {\frac{{\left( {\omega - \omega_{0} } \right) + n\omega_{g} }}{{\omega_{m} }}} \right)e^{{i\left( {t - \frac{{z_{out} }}{{v_{g} }}} \right)\left( {\omega - \omega_{0} } \right)}} d\left( {\omega - \omega_{0} } \right) $$

The expression in Eq. 6 is a super-resolution expression as the temporal spectrum is multiplied not by the temporal rectangular aperture, but rather by a synthetically extended temporal aperture. This aperture is continuously extended if we fulfill: $\omega_{m} \ge \omega_{g}$ and the super resolving factor i.e. the factor by which the synthetic aperture is larger in respect to the original time lens aperture is dependent on how many non-zero $B_{ - n} C_{n}$ coefficients we have in our grating. In the case of $\omega_{m} = \omega_{g}$ the super resolving factor equals exactly to the number of non-zero $B_{ - n} C_{n}$ coefficients.

When the above-mentioned condition, of n equal to –m does not happen, then we have in the integral of Eq. 5 a quadratic phase factor that multiplies the spectrum, and which is not canceled. If we observe again the expression of Eq. 5, we get that the quadratic phase equals to:

7 $$ \frac{{\beta_{2} z_{0} }}{2}\left( {\omega - \omega_{0} } \right)^{2} - \frac{{\beta_{2} z_{0} }}{2}\left( {\omega - \omega_{0} } \right)^{2} - \beta_{2} z_{0} \left( {\omega - \omega_{0} } \right)\omega_{g} \left( {n + m} \right) - \frac{{\beta_{2} z_{0} }}{2}\omega_{g}^{2} \left( {n + m} \right)^{2} $$

**Figure 3:**
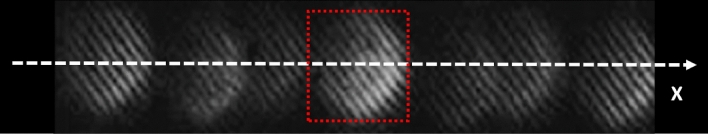
Experimental results of spatial-super-resolution with ghost images.

The first two terms cancel each other. The fourth term is a phase that does not depend on the spectral or the temporal coordinate and thus the interesting term is the third one. Having the temporal spectrum multiplied by such a term causes the temporal signal to be shifted in time by the amount of $\beta_{2} z_{0} \omega_{g} \left( {n + m} \right)$. Thus, this will generate ghost images^9^. Figure 3 illustrates this phenomenon in spatial imaging. This figure shows an experimental result for spatial super-resolution, in which spatial replications of the ghost images were accepted in addition to the super-resolved central replica. In the case of the temporal super-resolution, the same phenomenon will occur in the time domain.

Therefore, this will lead to a restriction on the input signal to be time-limited (equivalent to the field of view limitation in spatial imaging optics). The worst restriction on the temporal length of the signal is when (n + m) = 1 and then the signal is restricted to a temporal length of:

8 $$ \Delta \tau = \beta_{2} z_{0} \omega_{g} $$

Thus, if the temporal input signal fulfills this relation then the ghost terms that do not have the super-resolution condition, do not interact with the super-resolved term (the super-resolved reconstruction) for which (n + m) = 0.

### Temporal non-linear super-resolution

To obtain a super-resolution by the method proposed in this paper, the generation of short temporal pulses is necessary. As we proved in our mathematics, the final super-resolved outcome equals to the temporal resolution of the encoding sequence. Therefore, the super-resolution factor depends on how short the pulses are. Thus, it is essential to have an all-optical scheme capable of providing such short pulses.

To obtain a super-resolution by the proposed method, the temporal gratings in which we use should have temporal periodicity which is a low rate, i.e. not faster than the bandwidth of the time lens modulator. But each period of the temporal grating should have high temporal frequency (this is the meaning of having many terms of $B_{ - n} C_{n}$ non-zero). By having more non-zero Fourier series coefficients in the temporal grating we have a larger super-resolution factor. In spatial optics having gratings with high spatial frequencies is not a problem and those gratings are cheaper than having a high-quality imaging lens. But, in temporal optics both the temporal gratings as well as the time lens are realized with electro-optical modulators and super-resolution, in this case, aims to have low-frequency electronics which is cheaper than high-frequency circuits. Thus, to justify the usefulness of the proposed temporal super-resolution scheme, we need to show also a way of how to realize the encoding/decoding temporal gratings with low-frequency modulation electronics.

**Figure 4:**
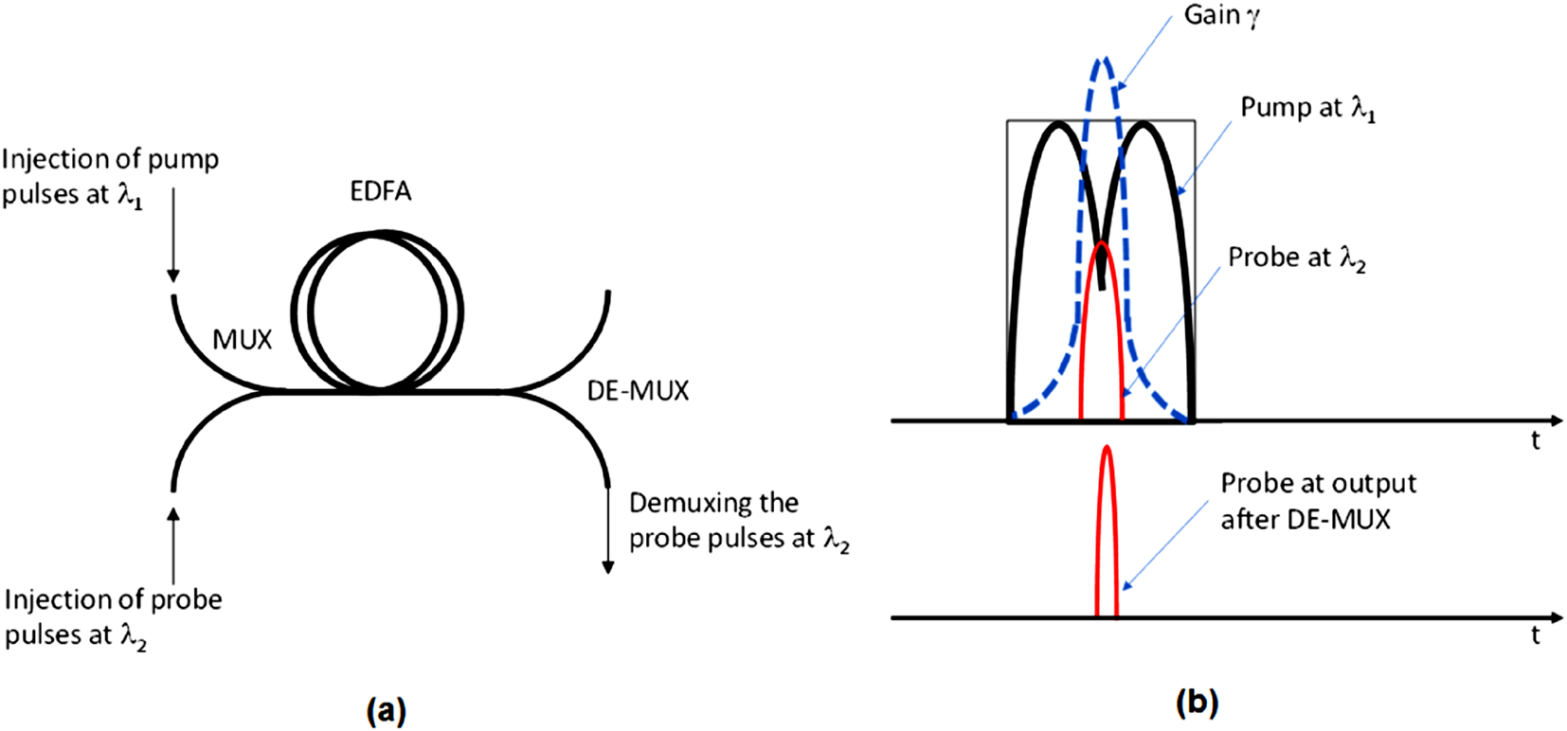
Realization of temporal gratings. (**a**) Schematic sketch of the proposed temporal super resolving imaging configuration. (**b**) Schematic illustration of the operation principle based on non-linearity and applied for the all-optical generation of the temporal gratings.

To do this, we aim to implement an all-optical way of realizing such gratings while bringing to the field of temporal optics terms taken from the field of non-linear (fluorescence-based) super-resolved spatial imaging. In spatial optics, the usage of nonlinear effect as the one existing in fluorescence motivated the development of various super resolving concepts, where the most common are the STED^32^, the PALM^33^ and the STORM^34^. In STED two light sources are used: the pump and the probe. The pump illuminates the fluorescent sample with a donut-like shape which narrows the point spread function (PSF) of the probe due to the stimulated emission-depletion effect (existing in fluorescence). This narrowing produces higher spatial frequencies and yields super-resolved image quality. What we propose here is to use a similar concept for realizing temporal grating having low-frequency temporal periodicity but high temporal frequencies within each of its temporal periods. The proposed idea is illustrated in Fig. 4a.

We propose to couple into one fiber two laser sources: probe and pump with slightly different wavelengths λ_1_ and λ_2_ respectively, where the pump is much stronger than the probe. We intend to use a 1-D donut-like pump signal (looks like a Gaussian with a dip in its center) and a Gaussian-like probe. The two signals are synchronized in time in such a way that the temporal position of the probe is exactly at the temporal position of the dip of the pump. The non-linear effect used in our case will be the non-linearity of a gain medium. We will insert those two waves into a gain medium (e.g. Erbium-doped fiber amplifier), and since the pump is much stronger than the probe, it sets very low gain at the small intensity values of the probe (temporal positions where the pump is strong) and high gain at the high values of the probe (temporal positions where the pump is weak). Thus, the pulse shape of the probe is being deformed and it narrowed in time while leads to an all-optical generation of higher temporal frequencies. A full description and mathematical analysis of this method and for the way it can be combined into our proposed super-resolution system are shown in the third section of the Supplementary document of this paper.

## Results

### Numerical simulation

**Figure 5:**
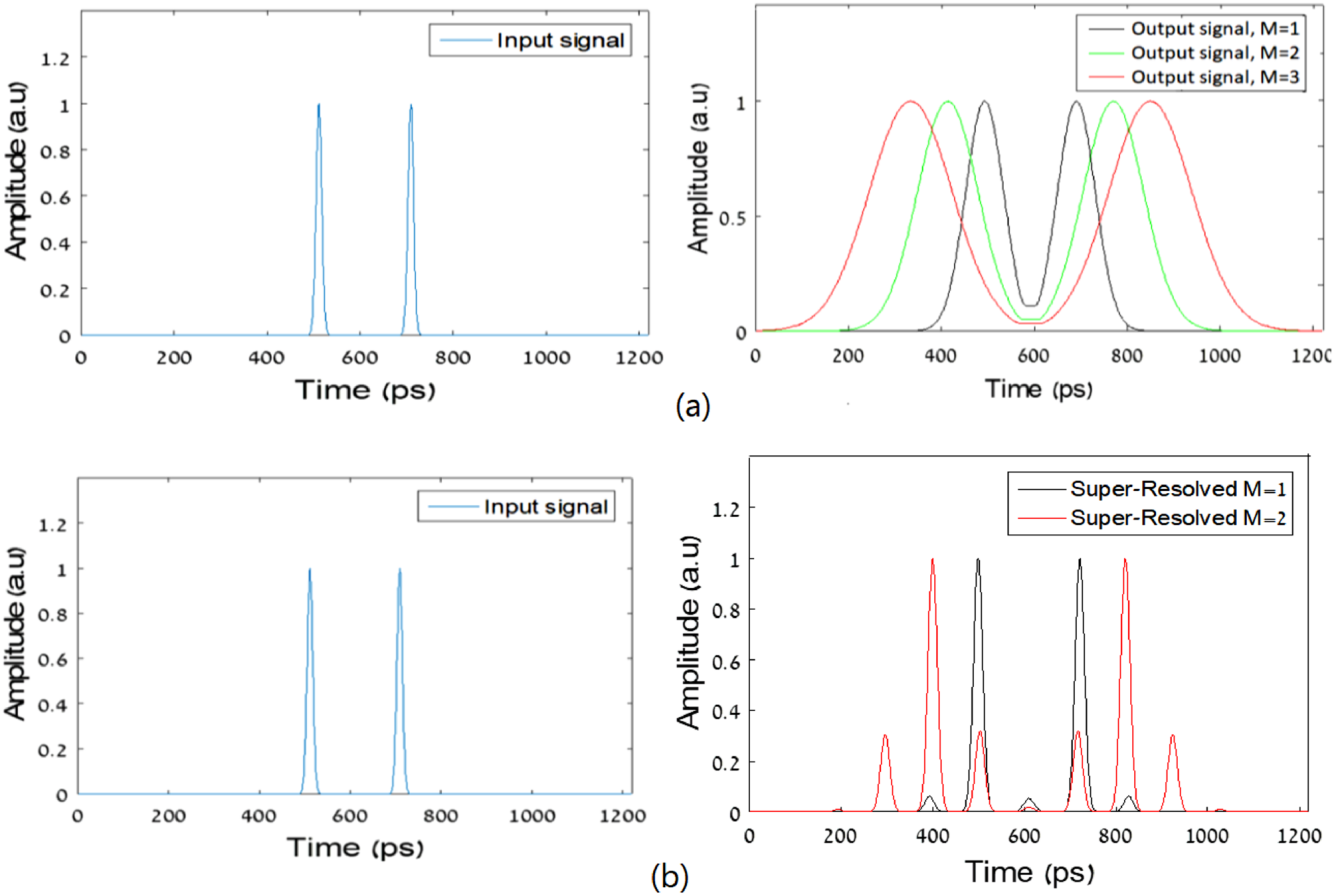
Numerical simulations: (**a**) Simulation of the basic temporal pinhole imaging system: input signal (left image) and output signals for three different temporal magnifications (right image). (**b**) Simulation of the super-resolved scheme: input signal (left image) and super-resolved signal (right image).

To validate the mathematical theory presented in this manuscript, we have constructed a MATLAB based simulator. In our simulations, we intend to show first the basic temporal imaging concept and then to add our super resolving concept. The simulation results can be seen in Fig. 5.

Figure 5a shows the simulation results for the temporal imaging concept based on the temporal pinhole imaging method^21,22^, where in Fig. 5b we show the simulation results of the same system while we added our proposed super resolving scheme. Two 15 ps Gaussian pulses with a distance of 200 ps between of them were inserted to those two systems. In the simulations, we chose the next parameters: The temporal length for the pinhole imaging pulse was 30 ps, the second-order dispersion coefficient was $\beta_{2} = 10.5 \times 10^{ - 24} {\text{s}}^{2} {\text{/km}}$ and the fiber lengths were z_1_ = 30 km and z_2_ = 30, 60 and 90 km for three different values for temporal magnifications M = 1, 2 and 3 respectively. Those different scaling factors are indeed seen in the results of Fig. 5a. For the super-resolution simulation, we used the same concept for temporal imaging and the same values of dispersion coefficient. The fiber lengths were z_0_ = 4 km, z_1_ = 30 and z_2_ = 30 and 60 km for two different values for temporal magnifications M = 1 and 2 respectively. A temporal grating with frequency $\omega_{g} = 10GHz$ was used. The results can be seen in Fig. 5b. We can clearly see that the low-resolution pulse is significantly wider in the time domain (85 ps) with respect to the super-resolved pulse (about 20 ps) which much more resembles the original pulse.

**Figure 6:**
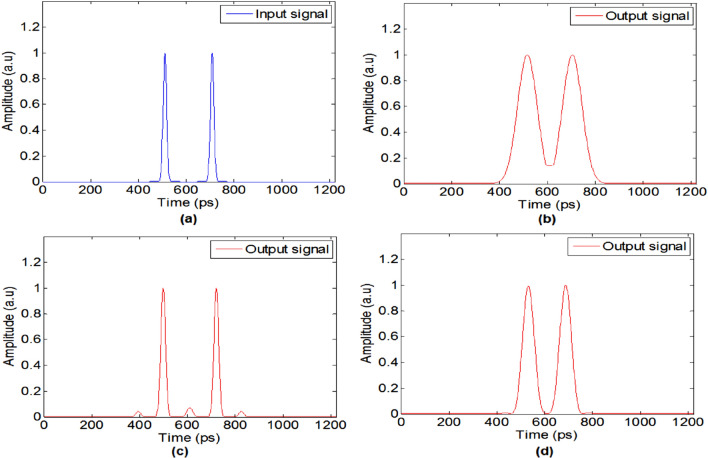
Simulation results: (**a**) two 15 ps input pulses. (**b**) Output signal for the pulse imaging system. (**c**) Output signal for super-resolution system with pulse train grating. (**d**) The output signal for super-resolution with grating consists of two sine waves.

As we mentioned above, the super resolving is dependent on how many non-zero $B_{ - n} C_{n}$ coefficients we have in our grating. Thus, a grating that consists of a summation of two sine waves can also improve the system temporal resolution by a factor of 2. Figure 6 shows simulation results of imaging of two 15 ps Gaussian pulses with a distance of 200 ps between of them. The figure shows the results for three different configurations of imaging systems: temporal pinhole imaging method, with a pulse width of 30 ps and magnification of M = 1, the proposed super-resolution system, with temporal grating with frequency $\omega_{g} = 10GHz$, and the proposed system with the simpler grating, that was generated by cascading two intensity modulators with frequencies modulation of 2.5 GHz and 7.5 GHz.

The simulation results show that the suggested grating improves the temporal resolution compare to the basic imaging setup. With this grating, each pulse in the output has temporal width of 55 ps, while in the basic imaging system we get 85 ps for each pulse, so this grating could serve us in demonstrating the resolution improvement cause by our proposed technique.

### Experimental results

To simplify the experimental setup, two separates experiments were performed, one for demonstrating the principle of the new super-resolution method, and another experiment that shows the ability to generate a train of temporally narrow pulses. In the first experiment, we used the simpler temporal grating that consists of a summation of two sine waves as we saw in the previous section, instead of using the short pulses train.

**Figure 7:**
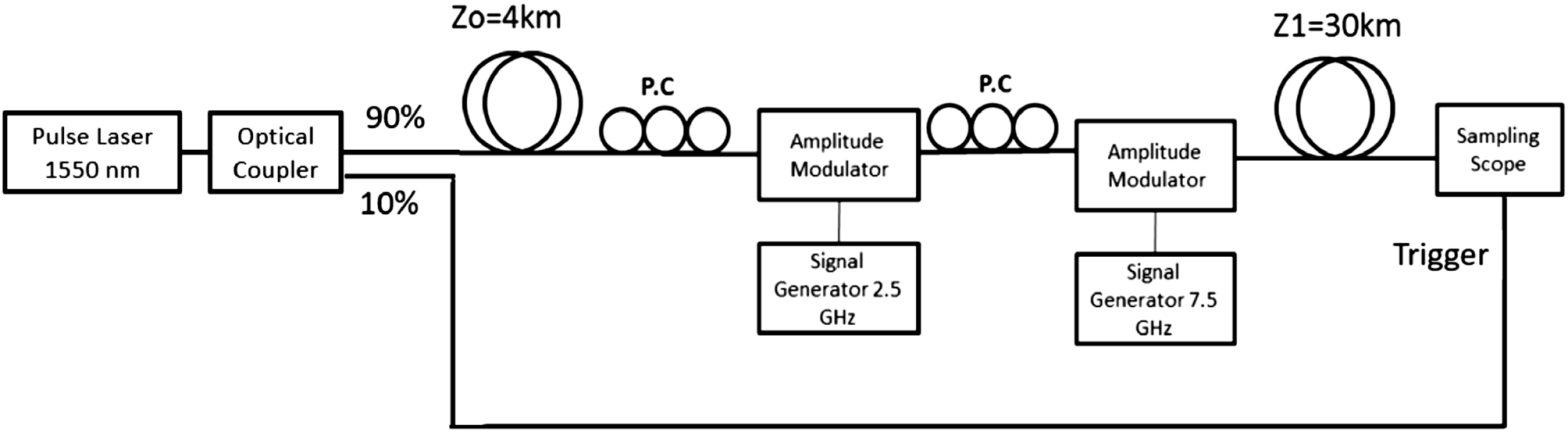
Schematic diagram of the experimental setup.

The purpose of the first experiment is to demonstrate the capabilities of the proposed system illustrated in Fig. 2. The first stage of this system, consists of dispersive elements and a temporal grating that are located before the time lens, should allow us to insert more information through the aperture of the temporal lens. Then, after the signal passes through the lens, we have another stage of dispersive elements and a temporal grating that allows us to obtain the undistorted spectral restoration of information. To simplify our setup, in this work only the first stage will be experimentally demonstrated, while the time lens and last stage of reconstruction will be numerically analyzed. Since the last stage of the system is very similar the first one, and since the realization of a time lens is not part of the novelty of our system, an experimental demonstration of the first stage of the system, together with numerical simulation of the other parts will be sufficient to demonstrate the capabilities of the system. The experimental part includes dispersive fiber, temporal grating and another dispersive fiber. The numerically analyzed part includes the rest of the system, i.e. temporal lens (temporal pinhole), dispersive fiber, additional temporal grating and negative dispersion fiber. A schematic diagram of the experimental setup can be seen in Fig. 7. A short pulse of about 12 ps was generated by using a pulse laser with a wavelength of 1550 nm. The pulse width was controlled by a tunable optical bandpass filter and it was split by an optical coupler with a coupling ratio of 90/10. The 10 percent of the signal was used as a clock and it was connected directly to the sampling scope (86100D Infiniium DCA-X Wide-Bandwidth Oscilloscope, Keysight Technologies) as a trigger. The other 90 percent of the pulse passes through 4 km single-mode fiber (silica, core diameter 8 μm, cladding diameter 125 μm, SILITEC fibers) with a dispersion coefficient of $\beta_{2} = 10.5 \times 10^{ - 24} {\text{s}}^{2} {\text{/km}}$ and then goes via two LiNbO_3_ optical intensity modulators, use Mach–Zehnder interferometer (IOAP-MOD9140-F-F-1 of JDS Uniphase), which function as temporal gratings. The pulse was modulated at frequencies of 2.5 GHz and 7.5 GHz. A Network analyzer (N5230A PNA-L of Agilent) was used as a signal generator to modulate the 7.5 GHz frequency and another signal generator (E4421B, ESG series of Agilent) was used for the 2.5 GHz modulation. Then the pulse was dispersed again through 30 km fiber with a dispersion coefficient of $\beta_{2} = 10.5 \times 10^{ - 24} {\text{s}}^{2} {\text{/km}}$. The output signal was detected by an optical sampling scope.

**Figure 8:**
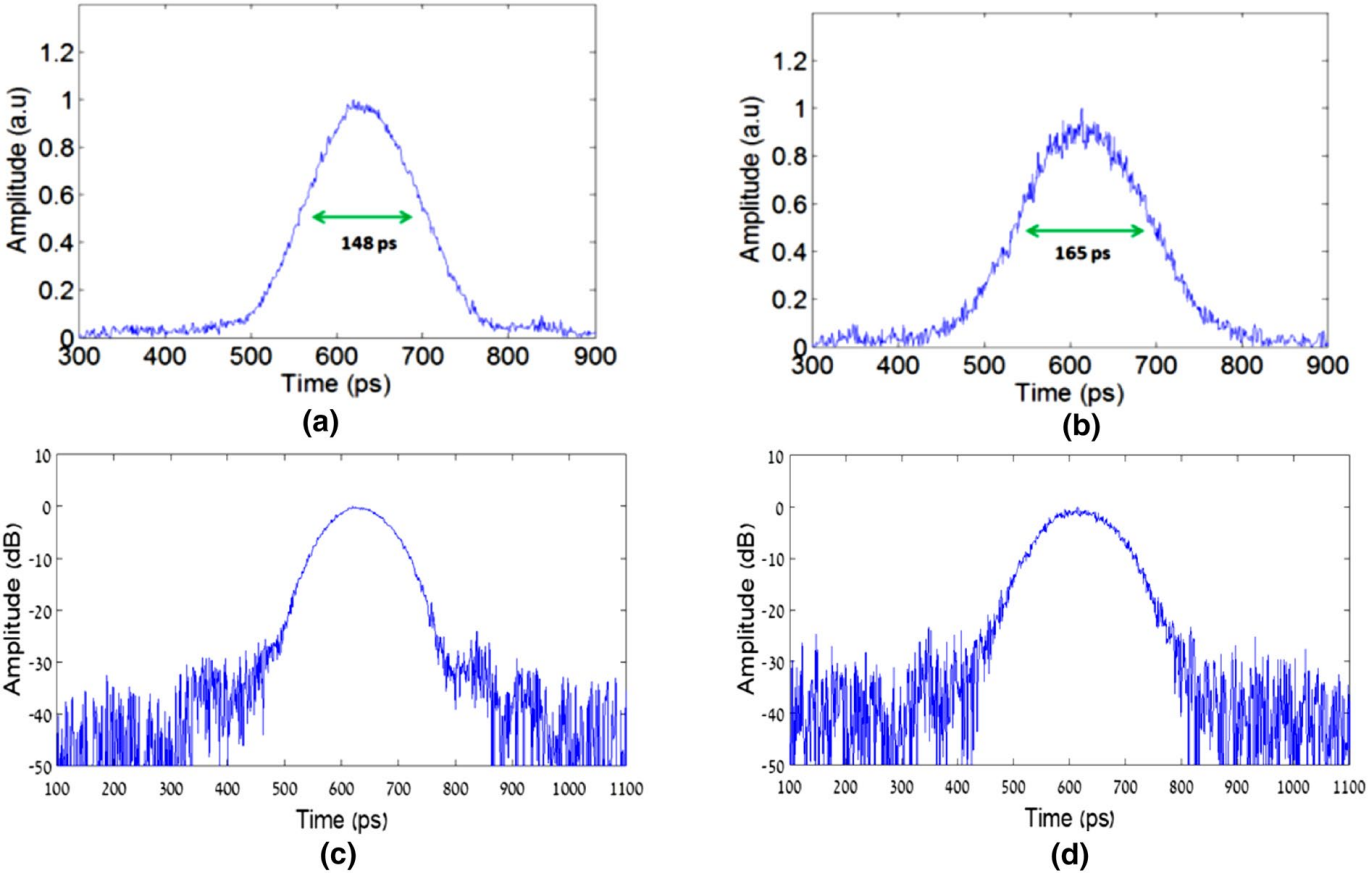
Measured signals in the output: (**a**) without modulation; (**b**) with modulation; (**c**) without modulation (vertical log- scale); (**d**) with modulation (vertical log- scale).

To investigate the accepted temporal resolution in our scheme, compared to the temporal resolution of the basic temporal imaging system, the output pulse was detected for two cases: with modulation (our scheme) and without modulation (standard imaging system). The detection was performed by a sampling scope with a temporal resolution of 1.22 ps. The detected pulses for both cases can be seen in Fig. 8.

**Figure 9:**
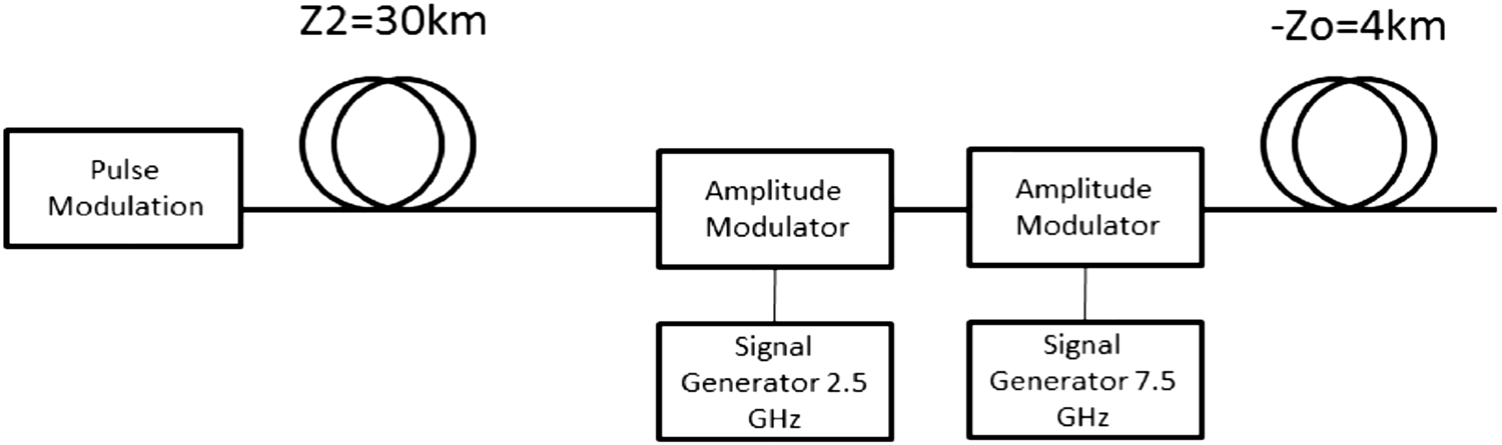
Schematic the digital analysis of the experimental results.

The modulated output signals were analyzed by the MATLAB code presented above. The pulse without modulation was inserted into digital pulse imaging (pulse width of 30 ps) and additional 34 km dispersion while the modulated pulse was inserted into digital analysis including pulse modulation for imaging (pulse width of 30 ps), additional dispersion of 30 km, temporal grating that consists of two sine waves, similar to the grating that was used in the experiment, and negative dispersion of 4 km. The dispersion coefficients for all the fibers in the simulation were $\beta_{2} = 10.5 \times 10^{ - 24} {\text{s}}^{2} {\text{/km}}$. A schematic diagram of the digital analysis that was performed to the modulated pulse can be seen in Fig. 9.

**Figure 10:**
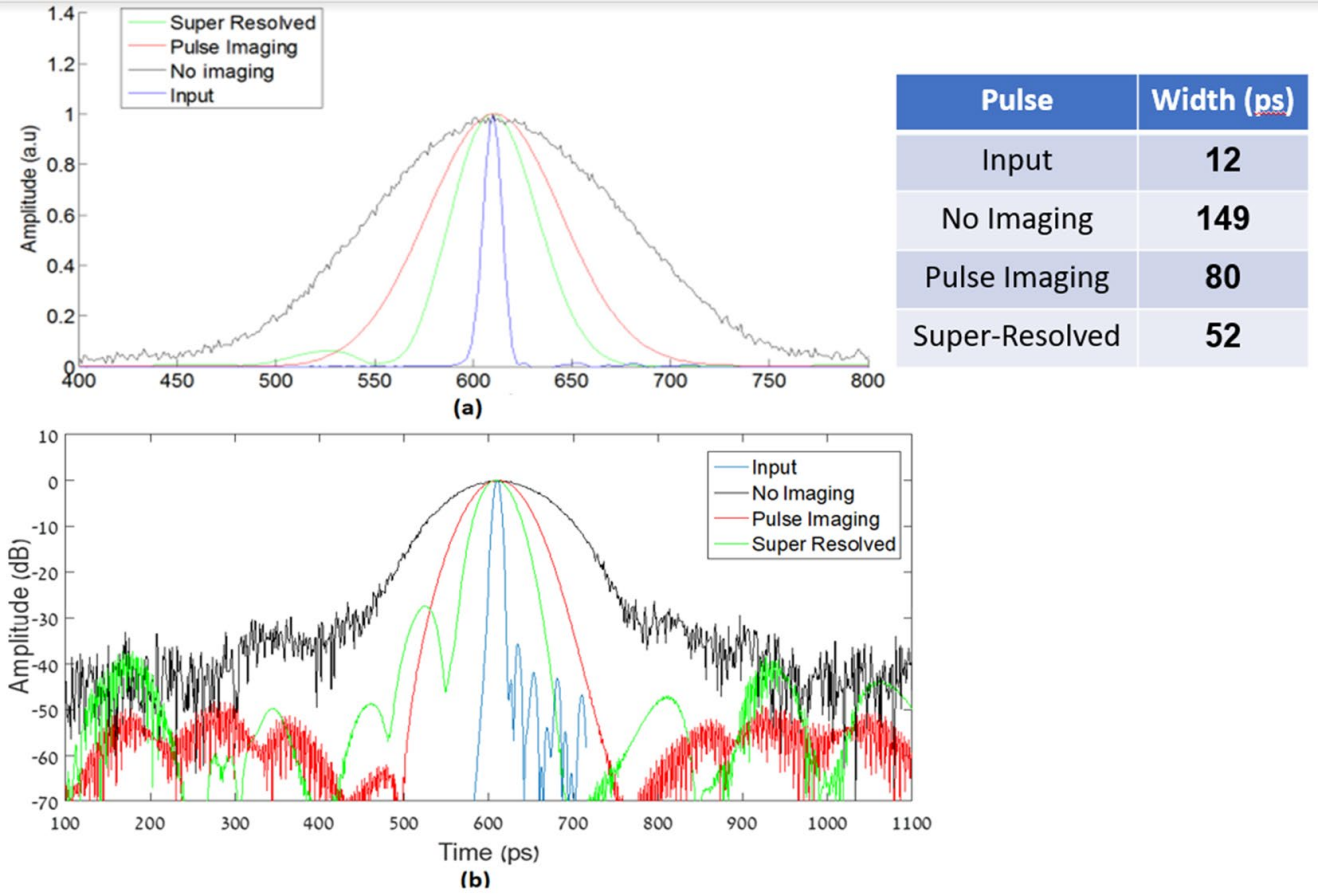
Experimental results: The output pulses for different configurations and their width in (**a**) vertical arbitrary unit (a.u) scale; (**b**) vertical log- scale.

The results of the digital analysis of the measured signals can be seen in Fig. 10.

**Figure 11:**
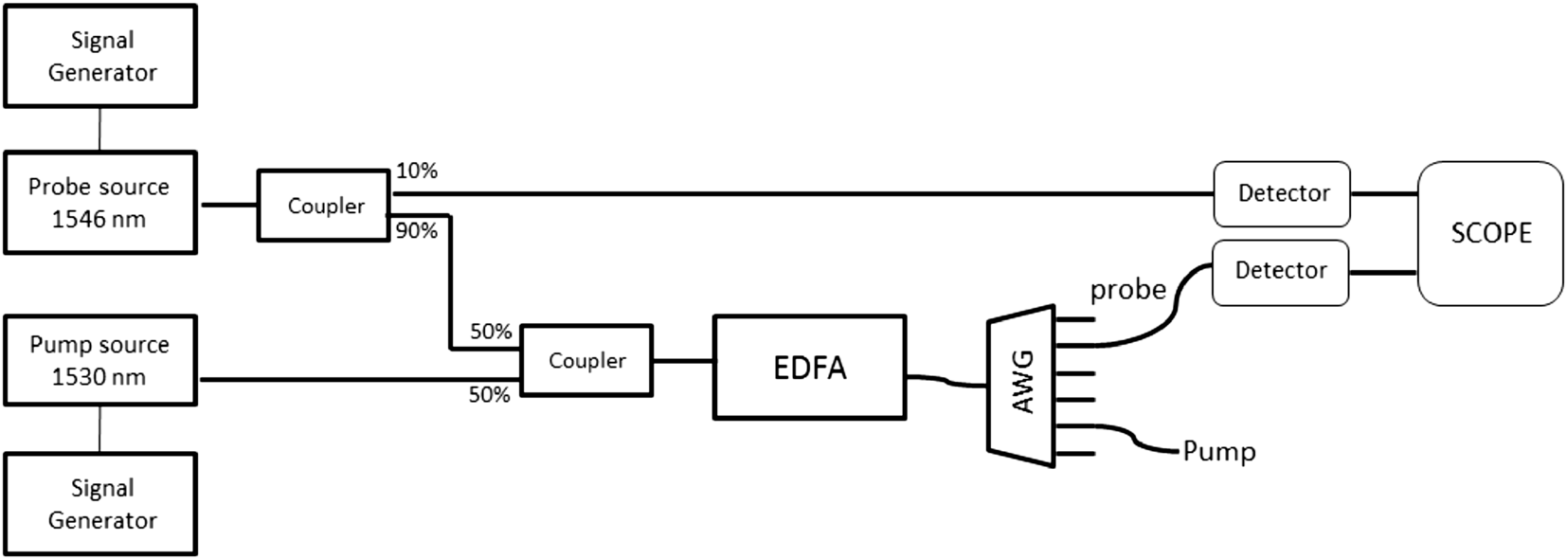
Experimental setup for shortening of optical pulses.

The second experimental setup was designed to demonstrate the ability to reduce the duration of optical pulses by using the method illustrated in Fig. 4. The experimental setup is shown in Fig. 11.

**Figure 12:**
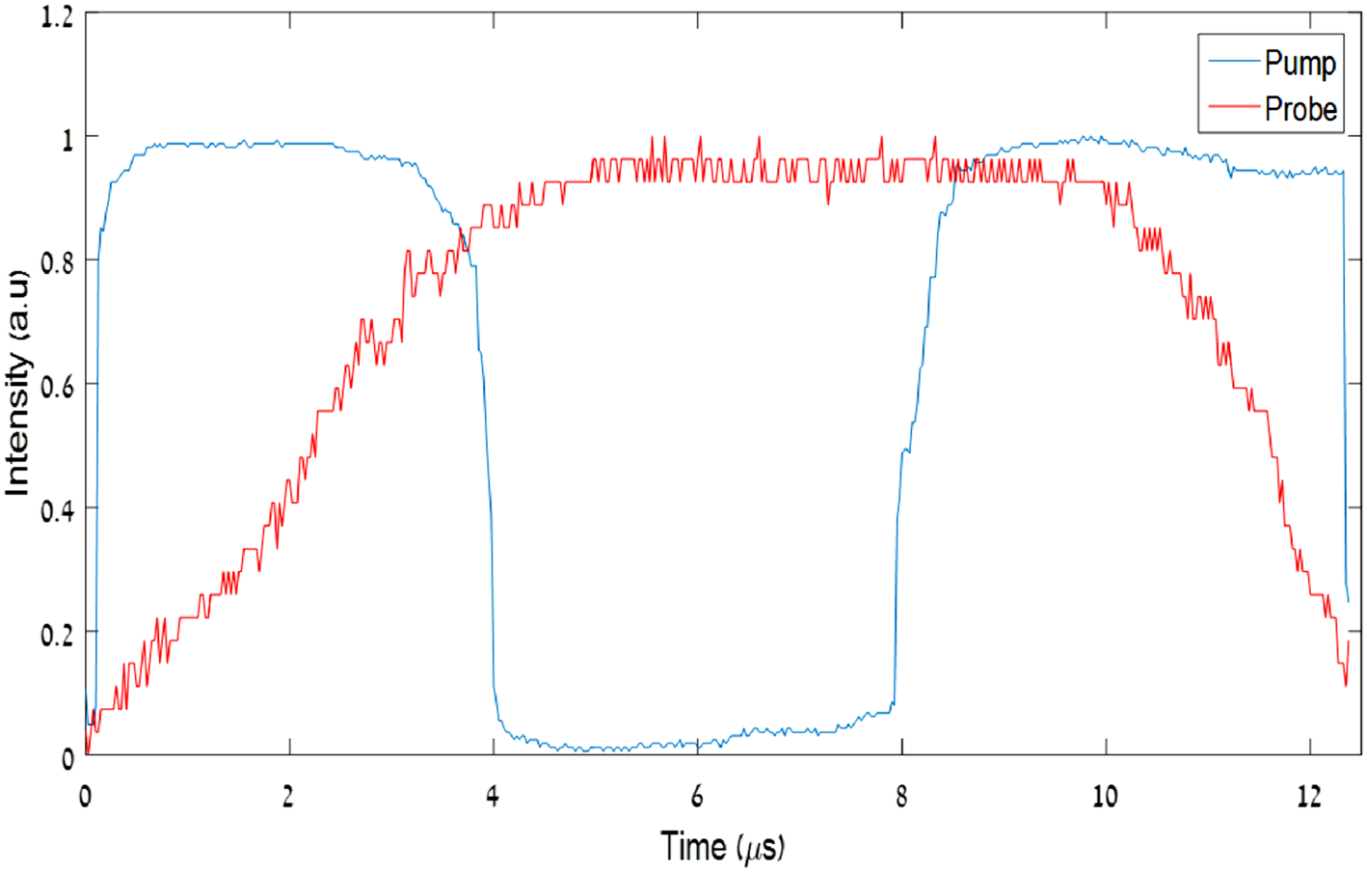
Pump and probe modulation*.*

A pump wave with a wavelength of 1530 nm and a probe with a wavelength of 1546 nm were modulated and were insert into one fiber by using an optical coupler. The shape modulation of each signal can be seen in Fig. 12.

**Figure 13:**
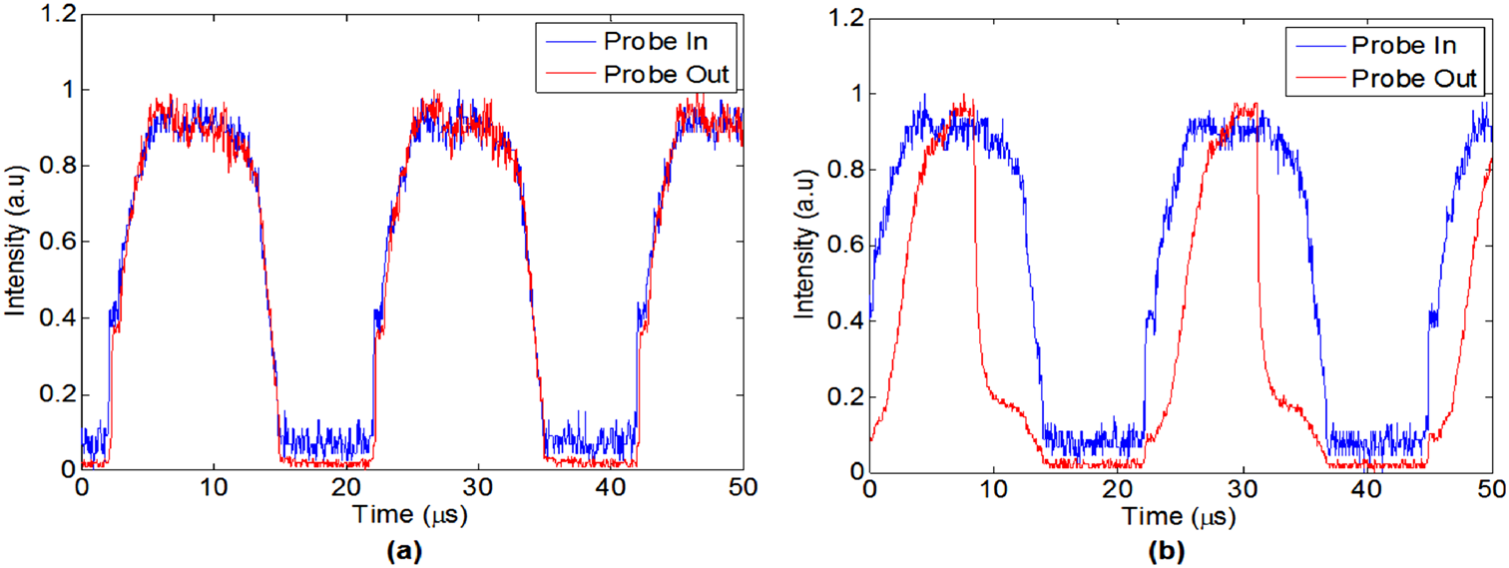
The input probe signal (blue line) compares to the output probe signal (red line) for two cases: (**a**) without using a pump; (**b**) by using a pump (left image).

The coupled signal was inserted into an EDFA. The intensities of the pump and the probe were chosen such that the pump intensity was seven times larger than the probe at the amplifier input. Then the pump and the probe were separated by using Arrayed Waveguide Grating (AWG), and the output probe was detected. The output probe compares to the input probe can be seen in Fig. 13, for the two cases, with and without a pump.

It can be seen that by using our proposed technique we could shorten the probe by factor 2. As we mentioned above our end goal is to generate a pulse train ,which its frequency of the should be lower or equal to the frequency of the time lens (see below Eq. 6), which its typical value is about 10 GHz for a time lens that is realized by an electro-optic phase modulator. The pulses duration depends on the desired temporal resolution and therefore it generally should be from the order of a few ps. Please note that in our experiment we demonstrated the principle for relatively long pulses, since EDFA is relatively slow in its time response. We used it in our experimental demonstration only because of its availability in our lab while the main goal is to demonstrate how gain and its associated non-linearity generate the temporal narrowing. There are much faster “gain” mechanisms in optics communication. For instance, the usage of SOA (semiconductor optical amplifiers) made of InGaAs has a much faster response time (few ps). There are nowadays technologies fabricating waveguides made out of InGaAs used for optical amplification. A similar experiment for shorter optical pulses can also be performed, so it is possible to generate the temporal grating required for our proposed temporal super-resolution imaging system.

## Discussion

Figure 8 shows the detected signals at the output of the experimental setup presented in Fig. 7 before the digital analysis. It can be seen that the measured signal for the case in which we applied the modulation is wider than the measured signal of the non-modulation case. Since multiplication by sine waves in the time domain is a convolution with delta functions in the Fourier domain, and since the output signal representing the Fourier domain (because we have long dispersive element between the temporal grating and the sampling scope), we should get replications of the signal, according to the modulation frequencies. Since the modulation frequencies are relatively low, the peaks of those replications will be very close to the main signal, so we see an expansion of our signal. This means that now we have more frequencies of the input signal per unit of time than the non-modulation case, so we could get a better reconstruction of the signal after passing the time-limited imaging pulse.

The results presented in Fig. 10 shows the output pulses for the different configurations after the digital analysis. It can be seen that by using pulse imaging, the output signal has a temporal length of 80 ps, i.e. it became narrower by a factor of 1.86 compared to the non-imaging case. For our proposed super-resolved scheme, the output pulse has a temporal length of 52 ps, i.e. it became narrower by a factor of 1.5 compared to the standard imaging process. Those results are very similar to the simulation results presented in Fig. 6. As we explained above, since we have a temporal grating that consists of two sine waves, we should get an improvement of the temporal resolution by a factor of 2, so our accepted results have also good agreement with the theory. In future works, a better temporal resolution may be achieved by using temporal gratings with more Fourier coefficients.

The small bumps on the left side of the experimental super-resolved signal stem from the small bumps of the input signal itself, as can clearly be seen in Fig. 10b. Those bumps were probably created following the use of the tunable optical bandpass filter used to control the width of the pulse. Since we used a temporal pinhole imaging method, those bumps were passed from the right side of the pulse in the input signal to the left side, which is the temporal equivalent to the inverted image accepted in a spatial pinhole camera.

It is important to note that for the reconstruction of the field (and not intensity) information is needed, i.e. amplitude and phase. In our numerical and experimental examination, we injected signals having only amplitude modulation and a constant phase. Indeed, when propagated through the system itself the signal gains phase but if the encoding pattern had no phase information then amplitude only decoding pattern would be able to fully reconstruct the super-resolved information. We have proved it before in our former research as e.g. seen in Ref.^35^. Thus, what is critical in order to obtain the super-resolved reconstruction is to have the encoding pattern be only amplitude pattern and be fully matched to the decoding pattern and then super-resolved reconstruction is feasible despite the system being non-ideal.

## Conclusion

In this paper, we have presented the conceptual methodology as well as the realization design of a novel temporal super-resolved imaging system. We showed that by using low rate electronics to implement a time lens modulator or used for the generation of a train of low-resolution temporal pulses (for the temporal encoding/decoding grating), one can transmit a temporal signal and recover it a temporal resolution higher than the bandwidth of the time lens and of the temporal gratings (thus, it could be done with simpler low-cost electronics). The proposed optical realization is based upon common optics communication components and does not require high-cost equipment.

Mathematical analysis, as well as numerical simulations, proved the proposed novel operation principle. Primary experimental were demonstrated resolution improvement by a factor of 1.5 based on the proposed method and additional experiment showed the ability to generate the temporal grating which is required for our new method. As part of our future research, we intend to experimentally validate the presented concept.

See Supplement 1 for supporting content.

## Supplementary information

Supplementary Information.
