# Supplementary material for: Fibers-based temporal super-resolved imaging
Source: Sci Rep. 2020 Oct 20;10:17750. doi: 10.1038/s41598-020-74879-z (PMC7576773; doi:10.1038/s41598-020-74879-z)
Supplement: Supplementary file 1 — Supplementary Information. [file 41598_2020_74879_MOESM1_ESM.pdf]

Fibers-based Temporal Super-Resolved Imaging: supplementary material

Sagie Asraf [a,*], Moti Fridman[a], Zeev Zalevsky[a,]

[a] Faculty of Engineering and the Institute for Nanotechnology and Advanced Materials Bar-Ilan University, Ramat-Gan, Israel, 52900.

*Corresponding author: Sagieas9@gmail.com

Abstract

This document provides supplementary information to “Fibers-based Temporal Super-Resolved Imaging”. In this document we present the full mathematical formulation of our new temporal super resolution configuration presented in the primary manuscript. In addition this document presents schematic description of the operation principle of the field of view (FOV) multiplexing in order to clarify the technique that proposed in the primary paper.

Temporal super resolution

We introduce now the mathematical model for our proposed temporal super resolution configuration. In the primary paper we saw that our proposed setup consists of several steps:

1. Propagation of the signal in a dispersive fiber with distance of z_0

2. Temporal grating

3. Another dispersive fiber with distance equal to $z_1 - z_0$.

4. Time lens which based on phase electro-optic modulator with radial frequency ω_m .

3. Another dispersive fiber with distance equal to $z_2 + z_0$.

4. Second temporal grating.

5. Propagation of the signal in a dispersive fiber with distance of z_0 , with negative dispersion coefficient.

We can formulate the propagation of the temporal signal in a dispersive fiber-based medium for distance of z0 as:

$$u\left(\omega_0,t,z_{0^-}\right)=u_0e^{i\omega_0 t-ik_{z,0}z_0}\int\tilde{U}\left(\omega-\omega_0\right)e^{i\frac{\beta_2 z_0}{2}(\omega-\omega_0)^2}e^{i(\omega-\omega_0)\left(t-\frac{z_0}{v_g}\right)}d\left(\omega-\omega_0\right) \quad \textbf{(S1)}$$

The above expression is multiplied by the first temporal grating $g_1(t)$ which can be decomposed according to Fourier series decomposition as:

$$g_1\left(t'\right)=\sum_n A_n e^{in\omega_g t'} \quad \textbf{(S2)}$$

Where ω_g is the radial frequency of this first grating and $t' = t - z_0 / v_g$. This results with:

$$u\left(\omega_0,t,z_{0^+}\right)=u_0e^{i\omega_0 t-ik_{z,0}z_0}\sum_n A_n\int\tilde{U}\left(\omega-\omega_0\right)e^{i\frac{\beta_2 Z_0}{2}(\omega-\omega_0)^2}e^{i\left(t-\frac{z_0}{v_g}\right)\left(\omega-\omega_0+n\omega_g\right)}d\left(\omega-\omega_0\right) \quad \textbf{(S3)}$$

Now we will mirror the effect of this temporal grating on the entrance point i.e. to propagate the electric field via the dispersion medium a distance of -z0 which yields:

$$u\left(\omega_0,t,0\right)=u_0e^{i\omega_0 t}\sum_n A_n\int\tilde{U}\left(\omega-\omega_0\right)e^{i\frac{\beta_2 Z_0}{2}(\omega-\omega_0)^2}e^{-i\frac{\beta_2 Z_0}{2}\left(\omega-\omega_0+n\omega_g\right)^2}e^{it\left(\omega-\omega_0+n\omega_g\right)}d\left(\omega-\omega_0\right) \quad \textbf{(S4)}$$

We will change our variables to: $\omega' = \omega - \omega_0 + n\omega_g$ and move to the position of the temporal aperture of the time lens (TL). For that a Fourier transform needs to be performed and we obtain (due to the analogy with space, the aperture of the lens multiplies the spectrum of the input signal (the object in spatial optics) that is to be imaged:

$$u\left(\omega_0,t,z_{TL}^-\right)=u_0e^{i\omega_0 t-ik_{zo}Z_{TL}}\sum_n A_n\tilde{U}\left(\omega'-n\omega_g\right)e^{i\frac{\beta_2 Z_0}{2}\left(\omega'-n\omega_g\right)^2}e^{-i\frac{\beta_2 Z_0}{2}\omega'^2} \quad \textbf{(S5)}$$

Now inserting the effect of the time lens which is limited in time (temporal imaging aperture length) as explained below and thus its time window may be modeled as: $rect(\omega'/\omega_m)$:

$$u\left(\omega_0,t,z_{TL}^+\right)=u_0e^{i\omega_0 t-ik_{zo}Z_{TL}}\sum_n A_n\tilde{U}\left(\omega'-n\omega_g\right)rect\left(\frac{\omega'}{\omega_m}\right)e^{i\frac{\beta_2 Z_0}{2}\left(\omega'-n\omega_g\right)^2}e^{-i\frac{\beta_2 Z_0}{2}\omega'^2} \quad \textbf{(S6)}$$

Now we will progress to the output (temporal image) plane and for that we need to perform another Fourier transform (following the analogy with spatial optics that was proven in the previous section, the relation between the aperture plane and the image plane is a Fourier transform) which yields:

$$u\left(\omega_0,t,z_{out}\right)=u_0e^{i\omega_0 t-ik_{z,0}z_{out}}\sum_n A_n\int\tilde{U}\left(\omega'-n\omega_g\right)rect\left(\frac{\omega'}{\omega_m}\right)e^{i\frac{\beta_2 Z_0}{2}\left(\omega'-n\omega_g\right)^2}e^{-i\frac{\beta_2 Z_0}{2}\omega'^2}e^{i\left(t-\frac{Z_{out}}{v_g}\right)\omega'}d\omega' \tag{S7}$$

Now we will propagate the electrical field via the dispersive medium of the fiber to the second grating $g_2\left(t\right)$. The propagation distance is z0 which yields:

$$u\left(\omega_0,t,z_{out}+z_0^{-}\right)=u_0e^{i\omega_0 t-ik_{z,o}\left(Z_{out}+Z_0\right)}\sum_n A_n\int\tilde{U}\left(\omega'-n\omega_g\right)rect\left(\frac{\omega'}{\omega_m}\right)\ldots$$
$$\ldots e^{i\frac{\beta_2 Z_0}{2}\left(\omega'-n\omega_g\right)^2-i\frac{\beta_2 Z_0}{2}\omega'^2+i\frac{\beta_2 Z_0}{2}\omega'^2}e^{i\left(t-\frac{Z_{out}+Z_0}{v_g}\right)\omega'}d\omega' \tag{S8}$$

Yielding:

$$u\left(\omega_0,t,z_{out}+z_{0^-}\right)=u_0e^{i\omega_0 t-ik_{zo}\left(z_{out}+z_0\right)}\sum_n A_n\int\tilde{U}\left(\omega'-n\omega_g\right)rect\left(\frac{\omega'}{\omega_m}\right)e^{i\frac{\beta_2 Z_0}{2}\left(\omega'-n\omega_g\right)^2}e^{i\left(t-\frac{Z_{out}+Z_0}{v_g}\right)\omega'}d\omega' \tag{S9}$$

The second grating, due to its periodicity, can also be decomposed according to Fourier series as:

$$g_2\left(t'\right)=\sum_m B_m e^{im\omega_g t''} \tag{S10}$$

Where $t''=t-(z_{out}+z_0)/v_g$ and the obtained result after modulating the signal with the second grating is:

$$u\left(\omega_0,t,z_{out}+z_{0^+}\right)=u_0e^{i\omega_0 t-ik_{z,o}\left(Z_{out}+z0\right)}\ldots$$
$$\ldots\sum_n\sum_m B_m A_n\int\tilde{U}\left(\omega'-n\omega_g\right)rect\left(\frac{\omega'}{\omega_m}\right)e^{i\frac{\beta_2 Z_0}{2}\left(\omega'-n\omega_g\right)^2}e^{i\left(t-\frac{Z_{out}+z0}{v_g}\right)\left(\omega'+m\omega_g\right)}d\omega' \tag{S11}$$

Now returning back to the output (temporal image) plane, gives us:

$$u\left(\omega_0,t,z_{out}\right)=u_0e^{i\omega_0 t-ik_{z,o}Z_{out}}\sum_n\sum_m B_mA_n\int\tilde{U}\left(\omega'-n\omega_g\right)\ldots$$

$$\ldots rect\left(\frac{\omega'}{\omega_m}\right)e^{i\frac{\beta_2Z_0}{2}\left(\omega'-n\omega_g\right)^2}e^{-i\frac{\beta_2Z_0}{2}\left(\omega'+m\omega_g\right)^2}e^{i\left(t-\frac{Z_{out}}{v_g}\right)\left(\omega'+m\omega_g\right)}d\omega' \tag{S12}$$

We will change our integration variables again back to: $\omega'=\omega-\omega_0+n\omega_g$ and obtain:

$$u\left(\omega_0,t,z_{out}\right)=u_0e^{i\omega_0 t-ik_{z,o}Z_{out}}\sum_n\sum_m B_mA_n\int\tilde{U}\left(\omega-\omega_0\right)rect\left(\frac{\omega-\omega_0+n\omega_g}{\omega_m}\right)\ldots$$

$$\ldots e^{i\frac{\beta_2Z_0}{2}\left(\omega-\omega_0\right)^2}e^{-i\frac{\beta_2Z_0}{2}\left(\omega-\omega_0+\omega_g\left(n+m\right)\right)^2}e^{i\left(t-\frac{Z_{out}}{v_g}\right)\left(\omega-\omega_0+\omega_g\left(n+m\right)\right)}d\left(\omega-\omega_0\right) \tag{S13}$$

SCHEMATIC EXPLANATION OF THE OPERATION PRINCIPLE

In this section we schematically explain how the FOV multiplexing super resolution operation principle works. The flow chart of computations includes several steps as follows:

1. **Propagation of z0 along our dispersion fiber.** This can be illustrated as seen in Fig. S1. Where we assume that the spectrum of our signal is the red Gaussian and the blue chirp function is the quadratic phase factor multiplying the spectrum of the signal due to the free space propagation.

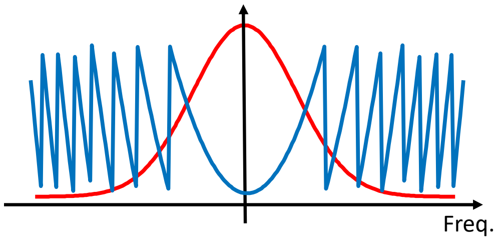

Fig. S1. Schematic illustration: Propagation of z0 along our dispersion fiber.

2. **Multiplication in time by an encoding grating.** Grating is a periodic function. Let us assume that it is a train of Dirac delta functions. This means that the Fourier transform of the grating function is also a train of Dirac delta functions (a property of a Fourier transform). Thus, the spectrum of the grating will look like illustrated in Fig. S2(a). Since multiplication in the time domain is a convolution in the Fourier domain, and a convolution between the function and a train of delta functions gives the replications of the function in the positions of the deltas. Thus, the spectrum of the signal after being multiplied by a grating in time (temporal grating) will look like it is seen in Fig. S2(b).

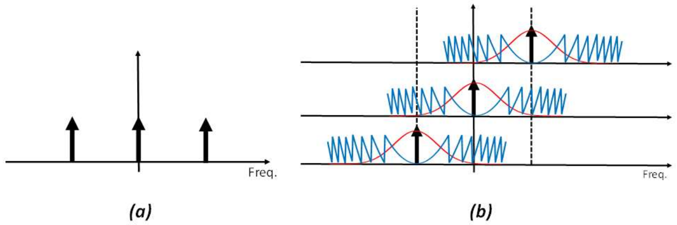

Fig. S2. Schematic illustration: Multiplication in time by an encoding grating. (a) The spectrum of the grating. (b) the spectrum of the signal after being multiplied by a grating in time.

3. **Propagation of -z0 along our dispersion fiber** (back to the input plane). This means that the spectrum that we have will be multiplied by an opposite quadratic phase factor (minus the previous phase factor). This negative or opposite quadratic phase factor is marked in green in the illustrative figure of Fig. S3.

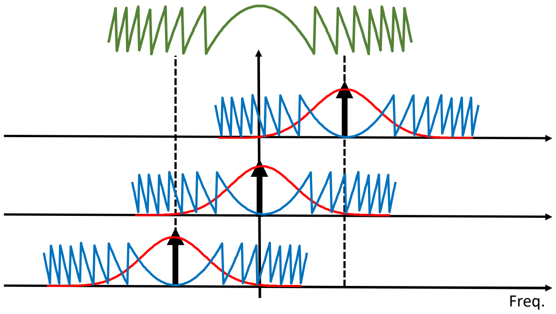

Fig. S3. Schematic illustration: Propagation of -z0 along our dispersion fiber.

We now need to add the phases (green and blue) for each one of the replicas. We need to remember that summation of two shifted opposite quadratic phases, results in a linear phase, as can be seen below where the result is a linear phase factor proportional to the spectrum coordinate ω , with proportion coefficient proportional to the relative shift $\delta\omega$ between the two opposite chirp functions, e.g.:

$$e^{\pi i a\left(\omega-\frac{\delta\omega}{2}\right)^2}\times e^{-\pi i a\left(\omega+\frac{\delta\omega}{2}\right)^2}=e^{-2\pi i a\delta\omega\,\omega} \quad \textbf{(S14)}$$

With a being a constant. Summation of two opposite non-shifted quadratic phases cancel each other. Thus, we have now the following spectrum where the blue lines depict the residual linear phase as seen in Fig. S4.

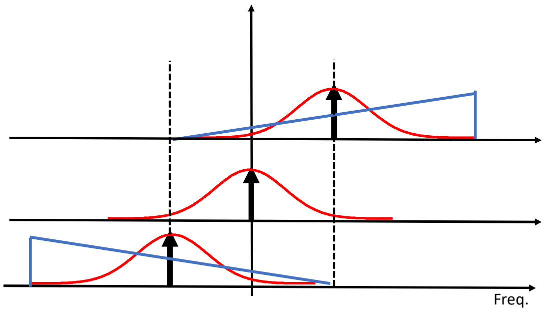

Fig. S4. Schematic illustration: Propagation of -z0 along our dispersion fiber.

4. **We go to the aperture plane.** This means that now we multiply the spectrum that we have by the aperture of the imaging lens which cuts out the high frequencies. Thus, now we have the spectrum as seen in Fig. S5.

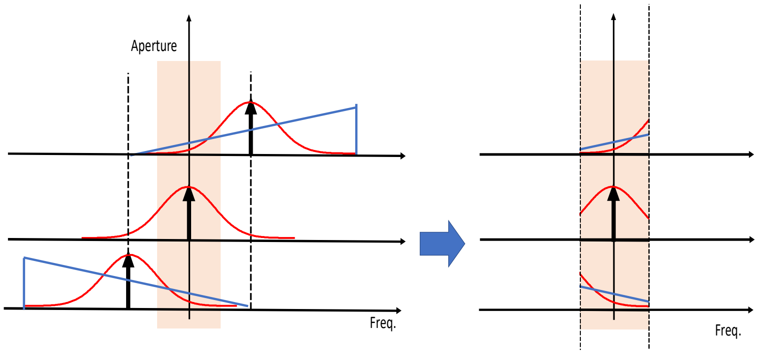

Fig. S5. Schematic illustration: The aperture plane.

5. **We go to the output plane**.
6. **We do another propagation of z0 along our dispersion fiber**, towards our decoding grating. This means that our spectrum is again multiplied by a quadratic phase factor, as seen in the left side of Fig. S6. Note that obviously the phase has a meaning only in spectral locations in which the amplitude is not zero. The amplitude is limited by the aperture, and thus what we really have is what is seen in the right side of Fig. S6.

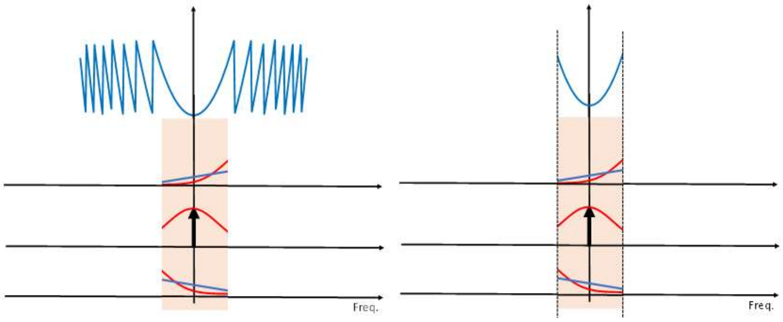

Fig. S6. Schematic illustration: A propagation of z0 along the dispersion fiber.

Note that the addition between a quadratic phase factor and a linear phase factor gives a shifted quadratic phase factor:

$$e^{\pi i a\left(\omega-\frac{\delta\omega}{2}\right)^2}\times e^{2\pi i a\delta\omega\,\omega}=e^{\pi i a\left(\omega+\frac{\delta\omega}{2}\right)^2} \quad \textbf{(S15)}$$

Thus, what we actually have in the spectrum looks like what is seen in Fig. S7.

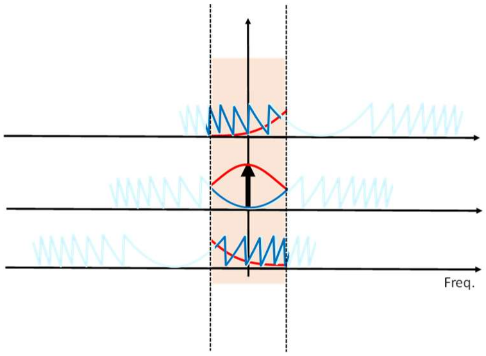

Fig. S7. Schematic illustration: A propagation of z0 along the dispersion fiber.

7. **We multiply in time by the decoding grating.** This means that as before in the spectrum we perform replications of the spectra and we have what is seen in Fig. S8.

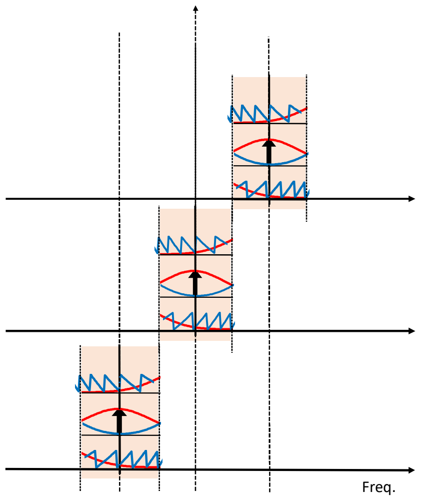

Fig. S8. Schematic illustration: multiply in time by the decoding grating.

8. **We perform propagation of -z0 along our dispersion fiber**, to go backwards to the output plane. Here is where we obtained the super-resolved reconstruction. As before this means that in the spectrum we multiply by an opposite quadratic phase factor (with minus) as seen in Fig. S9(a). As can be seen from the figure we have 9 spectral terms. The multiplication by the quadratic phase factor (green curve) will exactly cancel the phase of terms 3, 5, 7. In the other terms the multiplication will lead to a residual linear phase factor. The linear phase factor in spectrum generates the ghost images since linear spectral phase means shift in the time domain. The spectral terms of 3,5,7 in which the phase was exactly cancelled will give the super-resolved reconstruction. This is marked in purple color in Fig. S9(b).

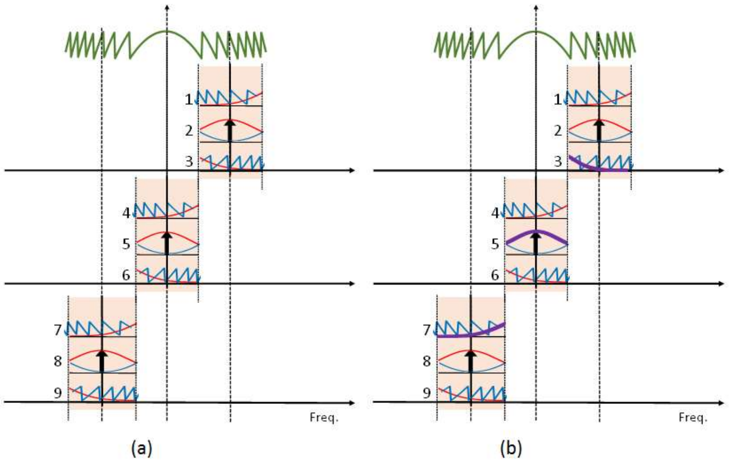

Fig. S9. Schematic illustration: Final step- propagation of -z0 along our dispersion fiber

By looking at the 3 purple curves it is clearly seen that it is the original spectrum having original width that was 3 times larger than the aperture of the temporal imaging lens, as seen in Fig. S10. We did not plot the other 6 spectral terms that

result in ghost images which due to their linear spectral phase lead to the limitation of the FOV as occurs in FOV multiplexing approach.

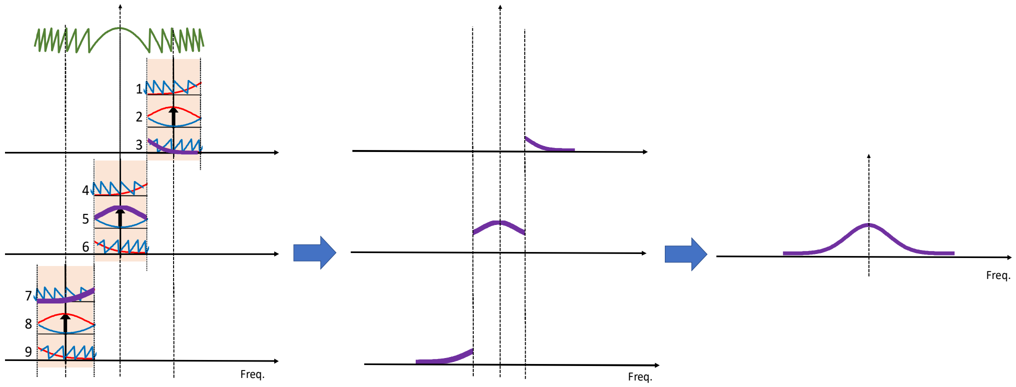

Fig. S10. Schematic illustration: Final step- propagation of -z0 along our dispersion fiber.

Temporal non-linear super-resolution

The proposed idea for generating the desired temporal grating is illustrated in Fig. S11(a).

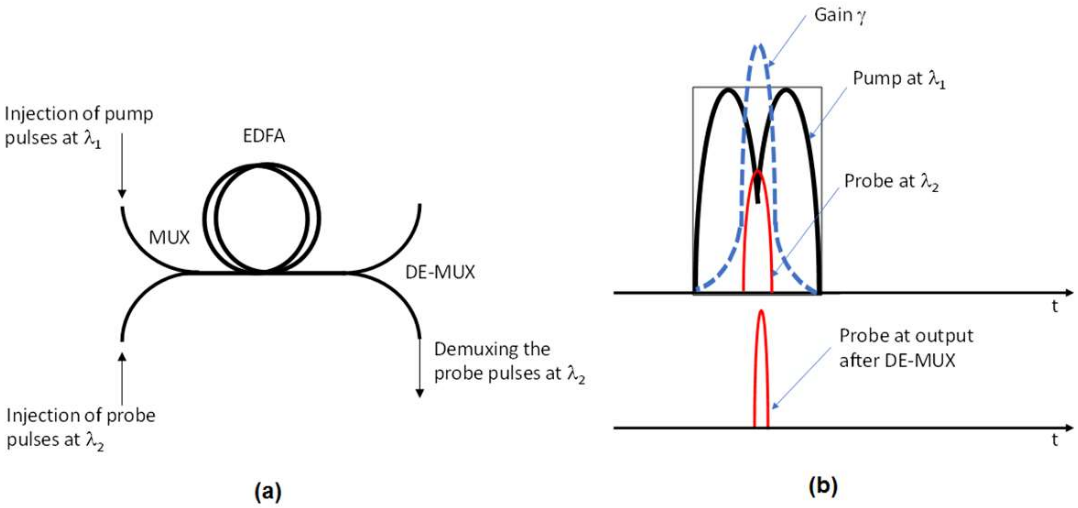

Fig. S11. Realization of temporal gratings. (a). Schematic sketch of the proposed temporal super resolving imaging configuration. (b). Schematic illustration of the operation principle based on non-linearity and applied for the all-optical generation of the temporal gratings.

The non-linear effect used in our case is the non-linearity of a gain medium (as happens in lasers). If fiber-based realization of the concept is applied, then the gain of Erbium-Doped Fiber Amplifiers (EDFA) can do the job (device commonly used in optics communication for doing all-optical amplification). We propose to connect to a fiber two sources- probe and pump. The pump is much stronger than the probe. Both are at slightly different wavelengths λ_1 and λ_2. The two wavelengths should be sufficiently close (spectrally) to each other for the gain

medium to have more or less the same amplification for both and yet they should be different enough so that we will be able to separate them using optics communication wavelength sensitive de-mux splitters.

The gain dependence on the intensity of photons going through the gain medium can be described as follows:

$$\gamma = \frac{\gamma_0}{1 + I_z / I_{sat}} \quad \textbf{(S16)}$$

Where γ_0 is called small signal amplification. I_z is the photons' intensity in the gain medium and I_{sat} is the intensity saturation value being typical for the specific gain medium. Since the intensity itself is also gain dependent, a non-linear dependency is obtained.

We intend to send a 1-D donut-like pump signal (looks like a Gaussian with a dip in its center) and a Gaussian-like probe. The two signals are synchronized in time in such a way that the temporal position of the probe is exactly at the temporal position of the dip of the pump. The pump is much stronger than the probe and thus it sets very low gain at the small intensity values of the probe (temporal positions where the pump is strong) and high gain at the high values of the probe (temporal positions where the pump is weak). Thus, the pulse shape of the probe is being deformed and it narrowed in time while leads to an all-optical generation of higher temporal frequencies. A schematic illustration is presented in Fig. S11(b).

Thus, if a train of such pulses is sent through our setup, in the output a train of temporally narrowed pulses will be generated. The periodicity of this train will be at low-frequency matched to the temporal imaging resolution of the time lens while in each period the temporal peaks are sharp and contain high temporal frequencies needed to realize the proposed super resolving operation principle previously described. Note that although a similar concept is implemented in a mode-locked laser, the proposed scheme is much simpler and cheaper for realization while it is using only low-cost conventional optics communication fiber-based equipment.

Now the question is how to couple (i.e. to generate a multiplication) between the optical train of pulses (i.e. the temporal grating) and the optical signal we aim to super resolve. To do this, the train of the narrow pulses will be combined (added via a coupler) to the optical input signal which we aim to super resolve and both will be passed again via another gain medium (fiber- based as EDFA). Again, we assume that the wavelength of our temporal signal is slightly different from the wavelength of the train of narrow pulses (which are at the wavelength of the original probe signal) such that later on they can be separated by wavelength sensitive de-mux splitter. We assume that the wavelength of our temporal information signal is λ_3 and that it is close to λ_1 and λ_2 (the wavelengths of the pump and the probe signals respectively) so that they all fall within the bandwidth of the gain medium. We will notate our temporal information signal by $s_0(t)$ and the temporal grating by:

$$g(t) = g_0 \sum_n rect[(t - 2\pi n / \omega_g) / \Delta t] \quad \textbf{(S17)}$$

Where Δt is the temporal width of the narrow pulses generated due to the non-linear interaction described above. Obviously$\Delta t \ll {}^{2\pi}/_{\omega_g}$.

By using Eq. S16 we can write the output intensity as:

$$I_z(t) = (s_0(t) + g(t)) \exp\left(\frac{z\gamma_0}{1 + (s_0(t) + g(t)) / I_{sat}} \right) \tag{S18}$$

Which gives:

$$I_z(t) = g(t) \exp\left(\frac{z\gamma_0}{1 + (s_0(t) + g(t)) / I_{sat}} \right) \Bigg|_{\lambda_2} + s_0(t) \exp\left(\frac{z\gamma_0}{1 + (s_0(t) + g(t)) / I_{sat}} \right) \Bigg|_{\lambda 3} \tag{S19}$$

After we perform the wavelengths sensitive splitting (de-mux) we are left only with the output signal at a wavelength of λ3. We assume that when the grating signal g(t) is zero then we have small signal amplification equal to γ_0, while when the grating signal is not zero then the gain is smaller and equals to:

$$\gamma = \frac{\gamma_0}{1 + (s_0(t) + g(t)) / I_{sat}} \tag{S20}$$

Thus, the signal at the output (the signal at a wavelength of λ3 only) will be equal to:

$$I_z(t) = s_0(t) e^{\gamma_0 z} - s_0(t) \Delta \sum_n rect\left(\frac{t - 2\pi n / \omega_g}{t} \right) \tag{S21}$$

Where we define that $\Delta \equiv e^{\gamma_0 z} - e^{\gamma z}$. The meaning of this result is that the input signal to our temporal super resolving system includes two terms. The first is just the information signal without being modulated by the temporal grating. This term will yield at the output of the temporal imaging system a low-resolution signal following the temporal imaging resolution of the temporal lens. The second term is the one that is multiplied by the temporal grating having the high temporal frequencies; this term will produce the super-resolution result proven in the previous section.

Thus, after combining the concepts described in the previous sections, the full temporally super-resolving system might be depicted as presented in Fig. S12.

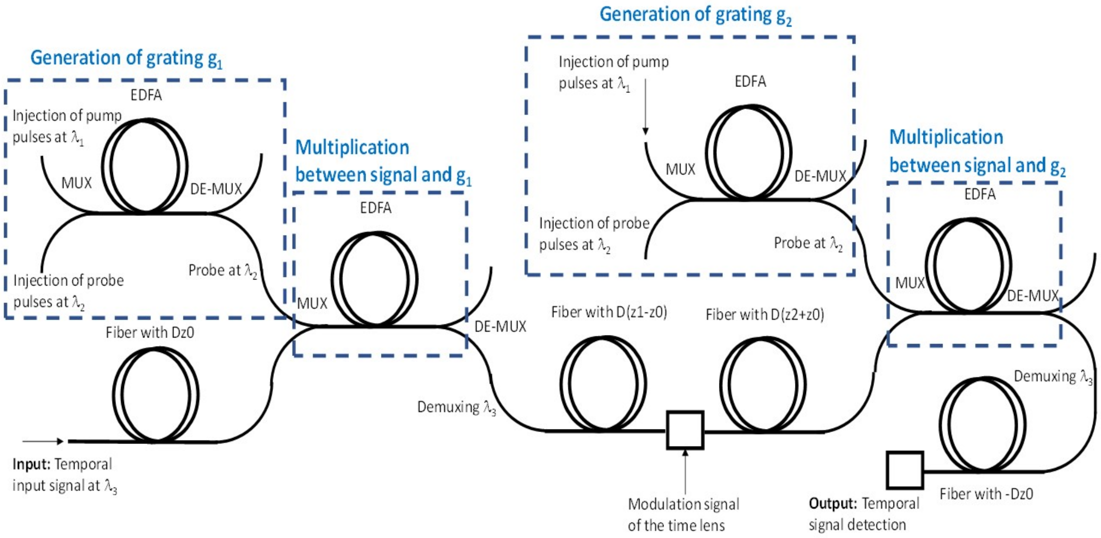

Fig. S12. Schematic illustration of the full temporal super-resolving imaging system.

Please note that EDFA is relatively slow in its time response. We used it in our experimental demonstration only because of its availability in our lab while the main goal is to demonstrate how gain and its associated non-linearity generate the temporal narrowing. There are much faster "gain" mechanisms in optics communication. For instance, usage of SOA (semiconductor optical amplifiers) made of InGaAs have much faster response time (few ps). There are nowadays technologies fabricating waveguides made out of InGaAs used for optical amplification.
